# Inflammation Determines the Pro-Adhesive Properties of High Extracellular D-Glucose in Human Endothelial Cells *In Vitro* and Rat Microvessels *In Vivo*

**DOI:** 10.1371/journal.pone.0010091

**Published:** 2010-04-08

**Authors:** Verónica Azcutia, May Abu-Taha, Tania Romacho, Marta Vázquez-Bella, Nuria Matesanz, Francis W. Luscinskas, Leocadio Rodríguez-Mañas, María Jesús Sanz, Carlos F. Sánchez-Ferrer, Concepción Peiró

**Affiliations:** 1 Departamento de Farmacología y Terapéutica, Facultad de Medicina, Universidad Autónoma de Madrid, Madrid, Spain; 2 Departamento de Farmacología, Facultad de Medicina, Universidad de Valencia, Valencia, Spain; 3 Center for Excellence in Vascular Biology, Department of Pathology, Brigham and Women's Hospital and Harvard Medical School, Boston, Massachusetts, United States of America; 4 Servicio de Geriatría y Unidad de Investigación, Hospital Universitario de Getafe, Madrid, Spain; 5 CibeRes CB06/06/0027 Carlos III Health Institute, Spanish Ministry of Health, Spain; University of Sheffield, United Kingdom

## Abstract

**Background:**

Hyperglycemia is acknowledged as an independent risk factor for developing diabetes-associated atherosclerosis. At present, most therapeutic approaches are targeted at a tight glycemic control in diabetic patients, although this fails to prevent macrovascular complications of the disease. Indeed, it remains highly controversial whether or not the mere elevation of extracellular D-glucose can directly promote vascular inflammation, which favors early pro-atherosclerotic events.

**Methods and Findings:**

In the present work, increasing extracellular D-glucose from 5.5 to 22 mmol/L was neither sufficient to induce intercellular adhesion molecule-1 (ICAM-1) and vascular cell adhesion molecule-1 (VCAM-1) expression, analyzed by flow cytometry, nor to promote leukocyte adhesion to human umbilical vein endothelial cells (HUVEC) *in vitro,* measured by flow chamber assays. Interestingly, the elevation of D-glucose levels potentiated ICAM-1 and VCAM-1 expression and leukocyte adhesion induced by a pro-inflammatory stimulus, such as interleukin (IL)-1β (5 ng/mL). In HUVEC, high D-glucose augmented the activation of extracellular signal-regulated kinase 1/2 (ERK 1/2) and nuclear transcription factor-κB (NF-κB) elicited by IL-1β, measured by Western blot and electromobility shift assay (EMSA), respectively, but had no effect by itself. Both ERK 1/2 and NF-κB were necessary for VCAM-1 expression, but not for ICAM-1 expression. *In vivo*, leukocyte trafficking was evaluated in the rat mesenteric microcirculation by intravital microscopy. In accordance with the *in vitro* data, the acute intraperitoneal injection of D-glucose increased leukocyte rolling flux, adhesion and migration, but only when IL-1β was co-administered.

**Conclusions:**

These results indicate that the elevation of extracellular D-glucose levels is not sufficient to promote vascular inflammation, and they highlight the pivotal role of a pro-inflammatory environment in diabetes, as a critical factor conditioning the early pro-atherosclerotic actions of hyperglycemia.

## Introduction

Vascular inflammation plays a pivotal role in the initiation and progression of the atherosclerotic plaque [1]. Indeed, the migration of circulating leukocytes from the blood to sites of extravascular injury is an early pro-atherosclerotic event mediated through a multistep adhesion cascade, initiated by the tethering of leukocytes to the endothelium, followed by weak, transient adhesive interactions manifested as leukocyte rolling, which leads to firm leukocyte adhesion and ultimately to transmigration through the vascular endothelium [2], [3]. Cell adhesion molecules (CAMs), including intercellular adhesion molecule-1 (ICAM-1) and vascular adhesion molecule-1 (VCAM-1), which are expressed by activated endothelial cells, play a crucial role in leukocyte adhesion and migration [2], [3].

Diabetes mellitus is characterized by a systemic pro-inflammatory environment, exhibiting enhanced basal and postprandial circulating levels of pro-inflammatory cytokines, including interleukin (IL)-1β, IL-6 and tumor necrosis factor-α (TNF-α) [4], [5]. An over-expression of pro-inflammatory CAMs has been reported in the cardiovascular system of animal models of diabetes [6], [7]. Furthermore, diabetic patients exhibit enhanced circulating levels of soluble ICAM-1 and VCAM-1 [8]–[10], which are considered to reflect vascular CAMs expression and represent prognostic markers of macrovascular complications and cardiovascular mortality [11].

Hyperglycemia, both basal and postprandial, has been identified over the years as an independent risk factor for cardiovascular diseases [4], [12], [13]. Indeed, sera from diabetic patients increase *in vitro* the adhesion of human monocytes and the expression of CAMs in human endothelial cells [14], [15]. It remains controversial, however, whether or not high D-glucose itself can stimulate such vascular pro-inflammatory mechanisms. Supporting a direct role of high D-glucose, Morigi et al. [14] first described enhanced VCAM-1 and ICAM-1 expression and leukocyte-endothelial adhesive interactions after incubating endothelial cultures for 24 h with 30 mmol/L extracellular D-glucose. Other studies have later on reported increased CAMs expression in human endothelial cells exposed to high D-glucose concentrations during time periods ranging from 24 h to 14 days [16]–[18], which has been attributed, at least in some cases, to hyperosmolarity [16].

Contrarily, an earlier work by Kim et al. [19] neither found induction of VCAM-1 and ICAM-1 nor enhanced adhesion of HL60 leukocytes by high D-glucose (25 mmol/L for 7–10 days) in human endothelial cells. More recently, Rasmussen et al. [15] suggested that the *in vitro* induction of CAMs in human endothelial cells by diabetic sera could not be solely attributed to high D-glucose concentrations, but more likely to the presence of serum cytokines. Additionally, both Cacicedo et al. [20] and Wada et al. [21] have demonstrated that high D-glucose alone does not induce the expression of genes encoding for VCAM-1 or ICAM-1 in human endothelial cells.

At present, the therapeutical approaches for preventing diabetes-associated cardiovascular diseases are mostly focused on tightly controlling hyperglycemia. In fact, some clinical trials in the 90's indicate that intensive blood-glucose control delays the onset and slows the progression of microvascular disease, both in type 1 and type 2 diabetes, although macrovascular complications are only prevented in a limited way after years of treatment [22]–[25]. Nevertheless, other recent trials with type 2 diabetic patients do not even show a beneficial effect of intensive glycemic control on the rate of cardiovascular events or deaths associated to atherosclerotic macrovascular disease [26]–[28]. Therefore, it has been now suggested that D-glucose is not the major mechanistic pro-atherosclerotic mediator in diabetes [29].

To clarify this point, it is necessary to carefully dissect the direct contribution of elevated D-glucose to the activation of early pro-inflammatory vascular events that initiate the atherosclerotic process. On the other hand, the potential interaction between D-glucose and pro-inflammatory cytokines, as two co-existing conditions in diabetes that may promote vascular inflammation, is still largely unknown and should be addressed. For this purpose, we have explored the respective contribution of both D-glucose at high concentrations and the pro-inflammatory cytokine IL-1β to endothelial CAMs expression and leukocyte trafficking, by combining both *in vitro* and *in vivo* approaches.

## Materials and Methods

### Ethics statement

The investigation conforms with the principles outlined in the Declaration of Helsinki. Experiments with human cells were reviewed and approved by the ethics committee of Universidad Autónoma de Madrid and Hospital Universitario de Getafe, and written informed consent was obtained from all donors. The investigation with animals conforms to the Guide for the Care and Use of Laboratory Animals published by the US National Institutes of Health (NIH Publication No. 85–23, revised 1996) and was approved by the ethics committee of Universidad de Valencia.

### Materials

Culture plastic ware was from TPP (Tragadingen, Switzerland). M199 and fetal calf serum were from Biological Industries (Beit-Haemek, Israel). Human recombinant IL-1β was purchased from Peprotech (London, UK), with an endotoxin level below 0.1 ng per µg. D-glucose was supplied by Serva (Heidelberg, Germany). Endothelial cell growth supplement, heparin, L-glucose, pyrrolidine dithiocarbamate, PD 98059 and, unless otherwise stated, all other reagents were purchased from Sigma Chemical Co. (St. Louis, MO).

### Cell isolation and culture

Human umbilical vein endothelial cells (HUVEC) were enzymatically isolated, as previously described [30], and cultured in M199 medium supplemented with 20% fetal calf serum (FCS), 25 µg/mL endothelial cell growth supplement (ECGS), 100 µg/mL heparin and antibiotics [30]. For experiments, HUVEC at passages 1–5 were used.

For adhesion under flow assays, HL60 leukocytes were obtained from American Type Culture Collection (ATCC; Rockville, MD) and grown in RPMI-1640 medium (Biowhittaker, Walkersville, MD) supplemented with 10% FCS and antibiotics.

### Flow cytometry

Confluent HUVEC monolayers were treated with different concentrations of D-glucose (5.5 and 22 mmol/L), either alone or in combination with increasing concentrations of IL-1β. The non-metabolizable analogue of D-glucose, L-glucose, was used as an osmotic control. After 18 h, the expression of VCAM-1 and ICAM-1 at the cell surface was measured by flow cytometry. In brief, HUVEC were gently detached with phosphate-buffered saline (PBS) containing 0.05% trypsin, fixed with 2% paraformaldehyde and blocked with PBS containing 3% bovine serum albumin (BSA) for 15 min. Cells were then suspended in PBS containing 0.5% BSA and incubated for 30 min with primary antibodies against VCAM-1 (clone IE5) or ICAM-1 (clone 6.5B5; Chemicon, Temecula, CA), both at a 1/100 dilution. Detection of primary antibodies was performed using an appropriate Alexa Fluor 488-secondary antibody (Molecular Probes-Invitrogen Corporation, Carlsbad, CA; dilution 1/250). Fluorescence was measured in a FACScan flow cytometer (Beckton-Dickinson, Franklin Lakes, NJ), and data analyzed using Cell Quest software (Beckton-Dickinson, Franklin Lakes, NJ).

The expression of CD11b/CD18 integrins was determined on human leukocytes in heparinized whole blood. Blood samples were obtained from buffy coats of four healthy donors by Ficoll Hypaque density gradient centrifugation. Samples (basal glucose concentration: 5.6±0.3 mmol/L) were incubated at 37°C with PBS or D-glucose (16.5 mmol/L to achieve a final concentration of around 22 mmol/L), either alone or in combination with IL-1β (5 ng/mL) for 18 h. Samples were then incubated for 20 min on ice in the dark with saturating amounts (10 µL) of the conjugated mAb anti-human-CD11b/CD18-FITC (clone ICRF 44; Serotec, Madrid, Spain). Red blood cells were lysed and leukocytes fixed using an automated EPICS Q-PREP system (Coulter Electronics, Hialeah, FL). Samples were run in an EPICS XL-MCL flow cytometer (Beckman-Coulter, Hialeah, FL) [31].

### Indirect immunofluorescence

ICAM-1, VCAM-1 and NF-κB were visualized in HUVEC by indirect immunofluorescence, accordingly to a previously described protocol [32]. Primary antibodies against VCAM-1 (dilution 1/250), ICAM-1 (dilution 1/25) or the NF-κB p65 subunit (dilution 1/100; Transduction Laboratories, Lexington, KY) were used, followed by incubation for 1 h at room temperature with an Alexa Fluor 488-conjugated secondary antibody (1/250). Cell nuclei were counterstained with 4′-6-diamidino-2-phenylindole (DAPI; Molecular Probes-Invitrogen). HUVEC were observed with an Eclipse TE300 epifluorescence microscope (Nikon, Tokyo, Japan).

### Nuclear extracts and electrophoretic mobility shift assay

HUVEC were exposed to the different treatments during 1, 4, 6 and 18 h and nuclear extracts were prepared as described before [32]. A commercial oligonucleotide (Promega, Madison, WI) encoding the NF-κB consensus sequence (5′-AGTTGAGGGGACTTTCCCAGGC-3′) was 5′-end labeled using [γ-^32^P]ATP and T4 polynucleotide kinase (Promega, Madison, WI) and purified using MicroSpin™ G-25 columns (GE Healthcare, Chicago, IL). For binding reactions, nuclear extracts (5 µg) were incubated on ice for 15 min in a reaction buffer [40 mmol/L HEPES (pH 7.0), 140 mmol/L NaCl, 5 mmol/L dithiothreitol, 10 µg/mL BSA, 0.01% Nonidet P-40, 4% Ficoll and 0.05 µg/mL poly(dI-dC).poly(dI-dC)]. After addition of the labeled oligonucleotide (∼50,000 cpm) the reaction mix was further incubated for 20 min at room temperature. For competition experiments a 100-fold excess of unlabeled doubled-stranded oligonucleotide was added to the binding reaction. DNA-protein complexes were resolved on 4% nondenaturing polyacrylamide gels in 0.5x TBE (45 mmol/L Tris-borate, 1 mmol/L EDTA, pH 8.0) at 4°C. Gels were dried and exposed to autoradiography at −80°C.

### Western blotting

Extracellular signal-regulated kinase 1/2 (ERK1/2) activation was determined by immunoblotting as previously described [32]. Polyclonal antibodies against both the phosphorylated (activated) and total forms of ERK 1/2 (Cell Signaling Technology, Inc., Danvers, MA; dilution 1/1,000) were used, followed by incubation with a horseradish peroxidase-conjugated secondary antibody (dilution 1/10,000; Chemicon, Temecula, California, USA). ERK 1/2 activity was expressed as the phospho-ERK 1/2:total ERK1/2 ratio.

### Flow chamber assays

The *in vitro* adhesion of HL60 to HUVEC monolayers was analyzed using a previously described live imaging flow model [33]. Briefly, HUVEC monolayers grown on glass coverslips were exposed for 18 h to 5.5 or 22 mmol/L D-glucose either alone or in combination with IL-1β (5 ng/mL). Coverslips were placed in a parallel plate flow chamber maintained at 37°C and HL60 leukocytes (1.5×10^6^ cells/mL) were drawn for 2 minutes across the monolayers at a flow rate of 0.26 mL/min, corresponding to at a shear stress of 0.5 dynes/cm^2^. Monolayers were visualized with an inverted microscope (Nikon TE2000, Nikon Inc., Melville, NY), and at least 5 fields were recorded during 10 seconds each using a phase contrast objective and VideoLab software (Ed Marcus Lbs, Newton, MA).

### Intravital microscopy

Non-diabetic male Sprague-Dawley rats (200–250 g) with a basal glycemia of 4.9±0.1 mmol/L (results from 5 animals) were employed. Animals were sedated with ether and intraperitoneally injected with 10 mL of either PBS alone, PBS with D-glucose (40 mg/kg), PBS with IL-1β (200 ng/kg), or PBS with D-glucose plus IL-1β. Parallel experiments were performed replacing D-glucose by L-glucose (40 mg/kg), which was used as an osmotic control.

After 18 h, the mesentery was exposed in preparation for intravital microscopy, following a previously described protocol [31]. Single unbranched mesenteric venules (25–40 µm in diameter) were selected, and the diameters measured on-line using a video caliper (Microcirculation Research Institute, Texas A&M University, TX). The number of rolling, adherent, and emigrated leukocytes was determined off-line during playback analysis of videotaped images.

### Immunohistochemistry

After the completion of the intravital microscopy measurements, the mesentery was isolated, fixed in 4% paraformaldehyde, dehydrated using graded acetone washes at 4°C, and embedded in paraffin wax for localization of ICAM-1 and VCAM-1, using a modified avidin and biotin immunoperoxidase technique as described previously [31]. Anti-rat-VCAM-1 (clone 5F10, kindly donated by Biogen Inc., Cambridge, MA) or anti-rat-ICAM-1 (clone 1A29, Serotec) monoclonal antibodies, or their isotype-matched control murine antibodies (UPC 10, IgG_2a_ and MOPC21 IgG_1_, Sigma Chemical Co.) were used. Positive staining was defined as a venule displaying brown reaction product.

### Statistical analysis

Results are expressed as mean±SEM from three to six independent experiments. Student's *t-test* was used for data points and one-way ANOVA with Fisher's post-test correction was used for curves. A *P* value ≤0.05 was considered statistically significant.

## Results

### ICAM-1 and VCAM-1 expression on HUVEC

**Figure 1:**
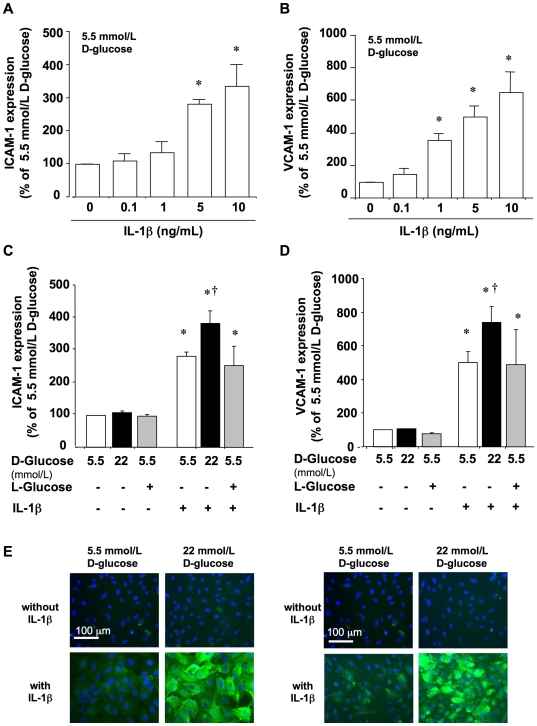
Effect of IL-1β and D-glucose on the expression of adhesion molecules in HUVEC. Cells incubated in a medium containing 5.5 mmol/L D-glucose were challenged for 18 h with IL-1β (0.1 to 10 ng/mL) and the levels of (A) ICAM-1 and (B) VCAM-1 were determined at the cell surface by flow cytometry. In another set of experiments, cells were incubated for 18 h in medium containing 5.5 or 22 mmol/L D-glucose in the presence or absence of IL-1β (5 ng/mL), after which (C) ICAM-1 and (D) VCAM-1 levels were quantified. L-glucose was used as an osmotic control. *P<0.05 versus 5.5 mmol/L D-glucose without IL-1β; †*P*<0.05 versus 5.5 mmol/L D-glucose with IL-1β. (E) ICAM-1 (left panel) and VCAM-1 (right panel) were visualized by indirect immunofluorescence in HUVEC cultured for 18 h in culture medium containing 5.5 or 22 mmol/L D-glucose in the presence or absence of IL-1β (5 ng/mL). Nuclei were counterstained in blue with 4′-6-diamidino-2-phenylindole (DAPI) (x400).

The mere elevation of extracellular D-glucose from 5.5 mmol/L up to 22 mmol/L did not alter the basal expression of both ICAM-1 and VCAM-1 in HUVEC after 18 h (107.05±4.4 and 104.5±3.7% of the expression observed with 5.5 mmol/L D-glucose, respectively. Figures 1C-1D). On the other hand, in HUVEC incubated in a medium containing 5.5 mmol/L D-glucose, the pro-inflammatory cytokine IL-1β enhanced the cell surface expression of both ICAM-1 and VCAM-1 in a concentration-dependent manner, with a sub-maximal effect observed at 5 ng/mL (Figures 1A-1B).

Interestingly, the stimulating effect of IL-1β (5 ng/mL) on ICAM-1 and VCAM-1 expression was potentiated when the extracellular D-glucose concentration was switched from 5.5 up to 22 mmol/L (Figures 1C-1D). These findings were also visualized by indirect immunofluorescence experiments, which confirmed the synergism between 22 mmol/L D-glucose and IL-1β on ICAM-1 and VCAM-1 expression, as well as the lack of effect of high D-glucose by itself on endothelial cell activation (Figure 1E). The interaction between high D-glucose and IL-1β was not attributable to hyperosmolarity, since it was not reproduced after replacing D-glucose by its non-metabolizable analogue L-glucose (Figures 1C-1D).

To assess whether the potentiating effect of 22 mmol/L D-glucose was restricted to IL-1β, we performed experiments in which IL-1β was replaced by tumor necrosis factor (TNF)-α (10 ng/mL). In HUVEC cultured in a 5.5 mmol/L D-glucose-containing medium, TNF-α significantly induced both ICAM-1 and VCAM-1 levels (1,037.21±182.82% and 156.38±17.45% non-stimulated HUVEC, respectively; *P*<0.05, results from four independent experiments performed in triplicate). When the glucose concentration was shifted up to 22 mmol/L, the expression of both ICAM-1 and VCAM-1 was further significantly enhanced to 1,364.52±106.99% and 206.86±14.45%, respectively (*P*<0.05 vs non-simulated HUVEC and *P*<0.05 vs matched TNF-α in 5.5 mmol/L D-glucose; results from four independent experiments performed in triplicate).

### CD11b/CD18 expression on human leukocytes

In human leukocytes, the expression of CD11b/CD18 integrins, involved in the leukocyte-endothelial interaction, was not significantly modified after 18 h of incubation in 22 mmol/L D-glucose (mean fluorescence intensity: 4.8±0.8 and 6.8±1.3 arbitrary units in 5.5 and 22 mmol/L D-glucose, respectively). However, when the cells were exposed to IL-1β (5 ng/mL), a marked induction of CD11b/CD18 was observed (mean fluorescence intensity: 13.0±3.2 arbitrary units, *P*<0.05 versus 5.5 mmol/L D-glucose), which was not further augmented in the presence of 22 mmol/L D-glucose (mean fluorescence intensity: 13.0±3.1 arbitrary units, *P*<0.05 versus 5.5 mmol/L D-glucose).

### ERK 1/2 and NF-κB activation in HUVEC and its role on ICAM-1 and VCAM-1 expression

**Figure 2:**
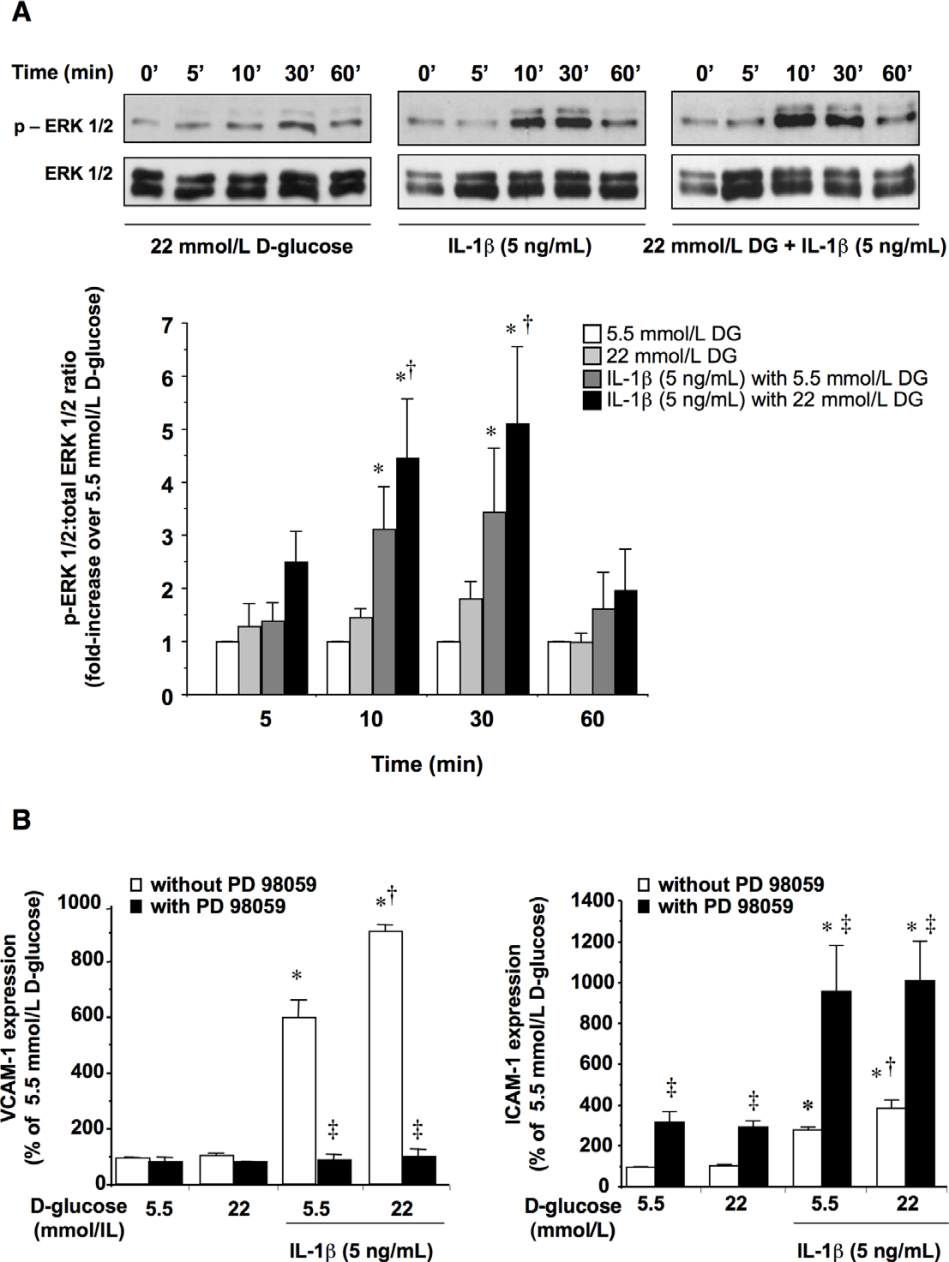
ERK 1/2 activation and its impact on ICAM-1 and VCAM-1 levels in HUVEC. (A) Cells were incubated in medium containing 5.5 mmol/L or 22 mmol/L D-glucose with or without IL-1β (5 ng/mL) for 5–60 min, after which ERK 1/2 activation was determined by Western blotting. Representative gels are shown on the top. (B) Involvement of ERK 1/2 in CAMs expression. HUVEC were cultured for 18 h in the above-described conditions. PD 98059 (30 µmol/L) was used as an inhibitor of ERK 1/2 activation. *P<0.05 versus 5.5 mmol/L D-glucose; †*P*<0.05 versus 5.5 mmol/L D-glucose in the presence of IL-1β; ‡*P*<0.05 versus matched treatment without PD 98059.

**Figure 3:**
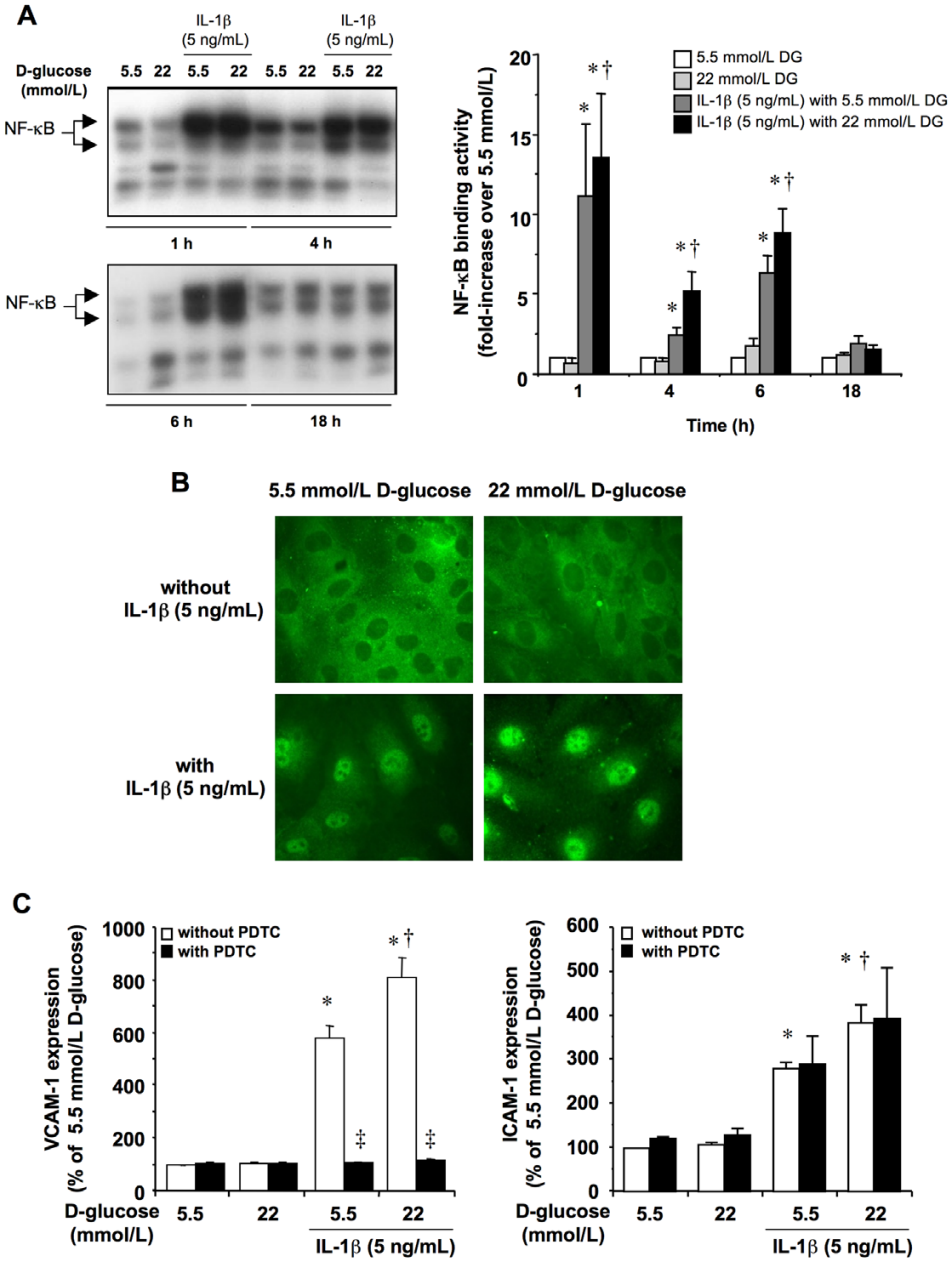
NF-κB activation in HUVEC and its impact on ICAM-1 and VCAM-1 levels. (A) HUVEC were incubated in medium containing 5.5 mmol/L or 22 mmol/L D-glucose in the presence or absence of IL-1β (5 ng/mL) during 1, 4, 6 and 18 h, after which NF-κB binding activity was quantified by EMSA. Representative EMSAs are shown on the left. (B) Translocation of NF-κB from cytoplasm to nucleus was visualized by indirect immunofluorescence in HUVEC cultured for 1 h as mentioned above (x1000). (C) Involvement of NF-κB in CAMs expression. HUVEC were cultured for 18 h in the above-mentioned conditions. PDTC (100 µmol/L) was used as NF-κB inhibitor. *P<0.05 versus 5.5 mmol/L D-glucose; †*P*<0.05 versus 5.5 mmol/L D-glucose with IL-1β; ‡ *P*<0.05 versus matched treatment without PDTC.

The incubation of HUVEC in 22 mmol/L D-glucose from 5 to 60 min did not result in increased ERK 1/2 activity, as compared with cells incubated in 5.5 mmol/L D-glucose (Figure 2A). Similarly, the exposure to high D-glucose did not result in NF-κB activation from 1 to 18 h after stimulation (Figure 3A). Conversely, IL-1β (5 ng/mL) in 5.5 mmol/L D-glucose activated ERK 1/2 after 10–30 min stimulation (Figure 2A) and triggered NF-κB binding activity from 1 to 6 h, returning to basal levels after 18 h (Figure 3A). The respective ERK 1/2 and NF-κβ activation by IL-1β was further enhanced when the cytokine was co-incubated with 22 mmol/L D-glucose (Figures 2A and 3A). The translocation of NF-κB from the cytoplasm to the nucleus was also visualized by immunofluorescence within the first hour of incubation (Figure 3B). Again, just enhancing extracellular D-glucose was not sufficient to promote NF-κB translocation, although it potentiated the activation of NF-κB elicited by IL-1β (Figure 3B).

To analyze the involvement of ERK 1/2 and NF-κB on ICAM-1 and VCAM-1 expression, HUVEC were treated with IL-1β (5 ng/mL), either alone or in the presence of the respective ERK 1/2 and NF-κB inhibitors PD 98059 (30 µmol/L) and PDTC (100 µmol/L) for 18 h. Both inhibitors abolished the induction of VCAM-1 elicited by IL-1β at any extracellular D-glucose concentration (Figures 2B and 3C). In contrast, PDTC was not able to modify the expression of ICAM-1 induced by IL-1β (Figure 3C), while PD 98059 up-regulated the expression of this CAM (Figure 2B).

### *In vitro* HL60 leukocyte adhesion to HUVEC

**Figure 4:**
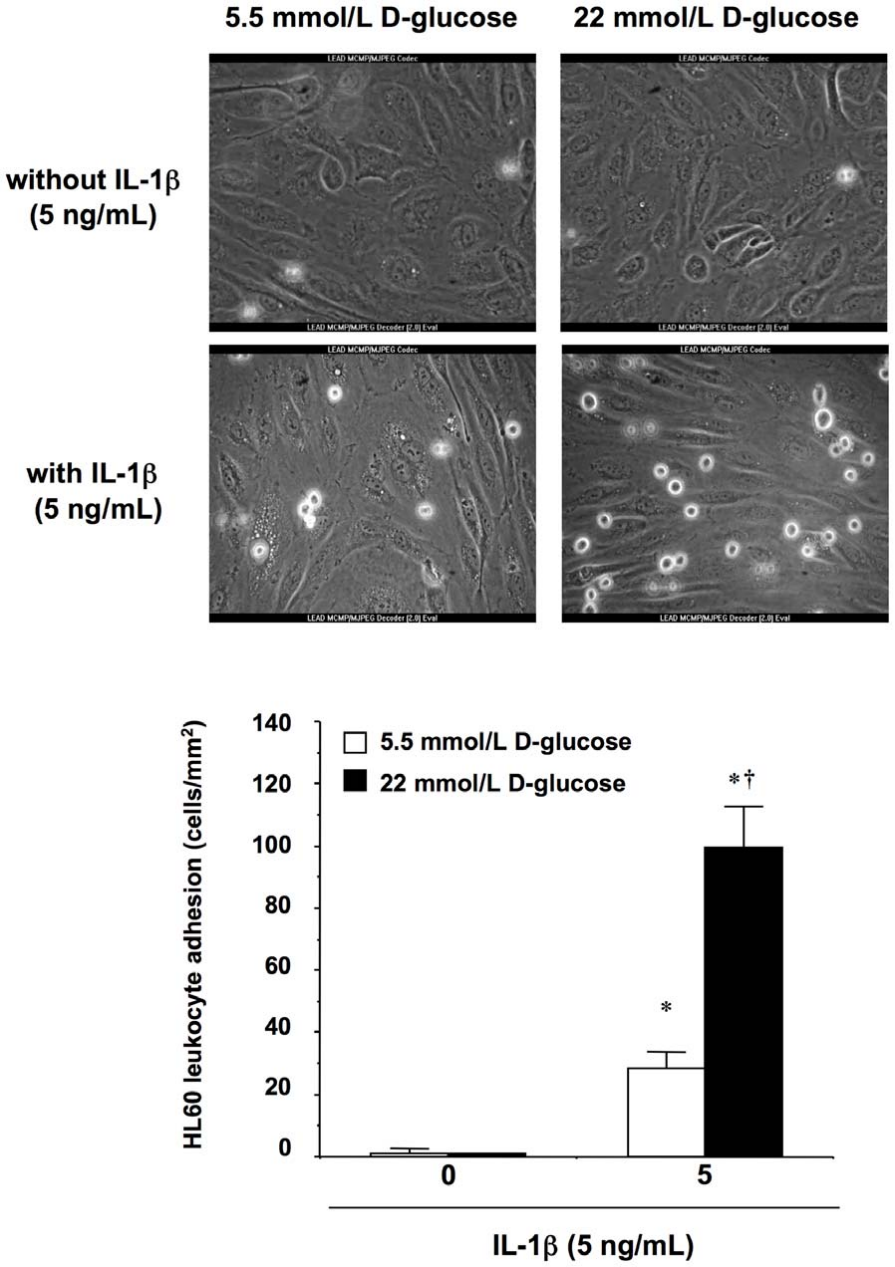
Adhesion of HL60 leukocytes to HUVEC under flow conditions *in vitro*. HUVEC monolayers were exposed to either 5.5 or 22 mmol/L extracellular D-glucose in the presence or absence of IL-1β (5 ng/mL) for 18 h prior to leukocyte perfusion. *P<0.05 versus 5.5 mmol/L D-glucose without IL-1β; †*P*<0.05 versus IL-1β in the presence of 5.5 mmol/L D-glucose. Representative micrographs showing HL60 adhesion to HUVEC monolayers are shown on the top (x200).

We next performed *in vitro* assays under flow conditions to investigate whether the synergism observed between high D-glucose and IL-1β on CAMs expression was paralleled by enhanced leukocyte adhesion to HUVEC monolayers. HL60 leukocyte adhesion was significantly enhanced when HUVEC were stimulated with IL-1β (5 ng/mL) in 5.5 mmol/L D-glucose medium for 18 h (Figure 4; Videos S1 and S3). This pro-adhesive effect of the cytokine was further enhanced by 3.7-fold when extracellular D-glucose was switched up to 22 mmol/L (Figure 4; Video S4). High D-glucose alone did not promote leukocyte adhesion to HUVEC (Figure 4; Video S2).

To assess whether the inhibition of either NF-κB or ERK 1/2 activation, which blocked VCAM-1 induction by IL-1β, could prevent the adhesion of HL60 leukocytes to endothelial cells adhesion, we performed additional experiments in which HUVEC were pre-incubated with PDTC (100 µmol/L) or PD 98059 (30 µmol/L). PDTC did not significantly modify HL60 adhesion induced by 5 ng/mL IL-1β (108.19±19.39 and 90.70±5.96% of IL-1β alone in 5.5 mmol/L and 22 mmol/L D-glucose medium, respectively; results from three independent experiments). For its part, PD 98059 only partially inhibited HL60 adhesion promoted by IL-1β (75.14±10.11 and 60.62±5.03% of IL-1β alone in 5.5 mmol/L and 22 mmol/L D-glucose medium, respectively; *P*<0.05 vs matched IL-1β alone; results from three independent experiments). In the absence of IL-1β, neither PD 98059 nor PDTC did affect HL60 adhesion, independently of the D-glucose concentration in the culture medium (data not shown).

### *In vivo* leukocyte trafficking

In order to extend the functional data obtained *in vitro* to an *in vivo* model, intravital microscopy was performed to examine leukocyte trafficking in the mesenteric microvasculature of Sprague-Dawley rats 18 h after the i.p. administration of a high dose of D-glucose (40 mg/kg) in the presence or absence of IL-1β (200 ng/kg).

**Figure 5:**
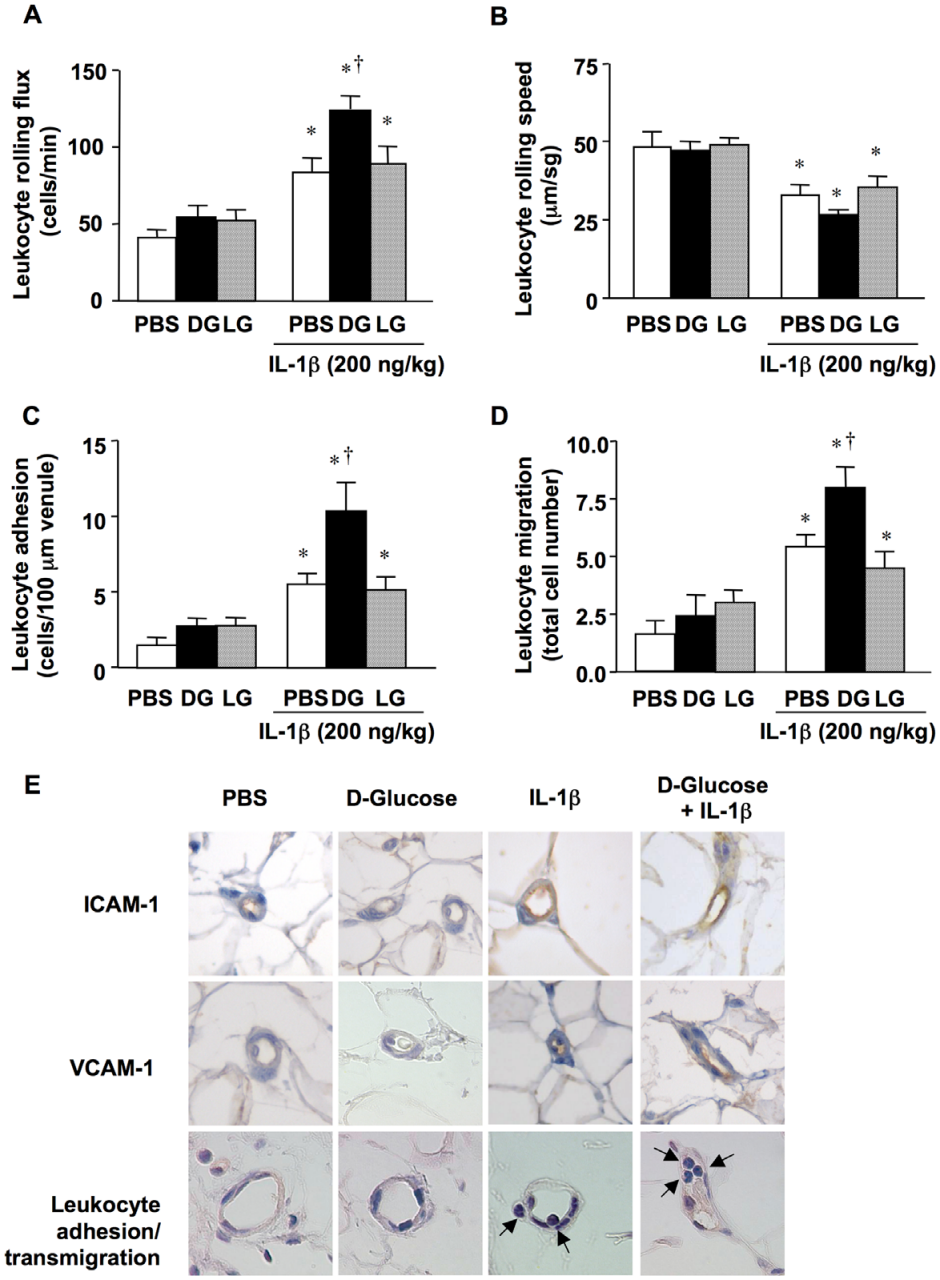
*In vivo* leukocyte trafficking in rat mesenteric post-capillary venules. Animals were i.p. injected with 10 mL of PBS alone (C) or supplemented with D-glucose (DG; 40 mg/kg), either in the absence or presence of IL-1β (200 ng/kg). L-glucose (LG; 40 mg/kg) was used as an osmotic control. After 18 h, (A) leukocyte rolling flux, (B) leukocyte rolling velocity, (C) leukocyte adhesion and (D) leukocyte migration were determined by intravital microscopy. *P<0.05 versus matched group without IL-1β; †*P*<0.05 versus IL-1β in PBS. Panel E shows representative photomicrographs showing ICAM-1 and VCAM-1 immunolocalization and leukocyte adhesion and transmigration in post-capillary venules. The brown reaction product indicates positive staining. Adhered and transmigrated leukocytes are marked with arrows in the right column. All panels are lightly counterstained with hematoxylin (x400).

When compared with control animals injected only with PBS, IL-1β-treated rats showed a significant increase in venular leukocyte rolling flux, adhesion, and emigration, as well as a concomitant decrease in venular leukocyte rolling velocity (Figures 5A-5D).

In those animals receiving only 40 mg/kg D-glucose, no changes in the above-mentioned trafficking parameters were observed (Figures 5A-5D). However, when IL-1β was co-injected with D-glucose, a clear increase in leukocyte rolling flux, adhesion, and migration was observed, as compared to IL-1β alone (Figures 5A, 5C and 5D). This latter effect was not due to hyperosmolarity, since L-glucose failed to mimic the potentiating effect of D-glucose (Figures 5A-5D).

At the time of the experiments, no significant differences in mean arterial blood pressure or venular shear rate were observed among the different treatments performed (Table 1).

**Table 1:** Hemodynamic parameters of Sprague-Dawley rats used for intravital microscopy studies.

Treatment	Mean arterial blood pressure (mm Hg)	Shear rate in venules (s^−1^)
PBS	108.8±2.6	525.4±42.7
D-glucose	112.6±3.9	493.9±50.0
L-glucose	114.6±4.0	469.5±50.4
IL-1β	113.4±4.6	502.8±27.0
IL-1β + D-glucose	115.3±3.9	477.5±66.9
IL-1β + L-glucose	108.8±5.5	500.8±39.2

Parameters (mean ± SEM) were measured 18 h after the i.p. injection of 10 mL of PBS alone (*n* = 6), or containing D-glucose (40 mg/kg; *n* = 5), L-glucose (40 mg/kg; *n* = 5), IL-1β (200 ng/kg; *n* = 7), D-glucose plus IL-1β (*n* = 6) or L-glucose plus IL-1β (*n* = 5). No significant differences were found in mean arterial blood pressure or shear rate in venules between any of the different treatments performed.

### Immunohistochemical and histological studies

Immunohistochemical studies of the mesenteric tissue obtained from PBS-treated animals revealed a weak staining for ICAM-1, which was not modified in animals receiving only D-glucose (Figure 5E). Additionally, no adhered or emigrated leukocytes were observed in microvessels from PBS- or D-glucose-treated animals (Figure 5E). IL-1β injection enhanced positive staining for both CAMs, and leukocyte adhesion and transmigration through the endothelial layer, especially in animals co-injected with D-glucose (Figure 5E).

## Discussion

The data presented herein do not support a role for high extracellular D-glucose alone in promoting pro-inflammatory endothelium-leukocyte interactions. In this context, just increasing D-glucose concentration up to 22 mmol/L, which is twice the plasma concentration considered to be indicative of diabetes after the oral glucose tolerance test (11.1 mmol/L) [34], did not modify CAMs expression or the adhesion of HL60 leukocytes to HUVEC monolayers. Furthermore, the elevation of extracellular D-glucose did not affect the expression of CD11b/CD18 integrins in leukocytes, which are pivotal leukocyte molecules for adhesion and migration [35]. Consistently, the increased monocytic expression of adhesion receptors observed in type 2 diabetic patients correlates with body mass index or serum markers of inflammation and only to a lesser extent with glycemic levels [5].

It is well established that pro-inflammatory cytokines are key molecules in mediating leukocyte adhesion and transendothelial migration [2]. Accordingly, we observed that IL-1β promoted endothelial ICAM-1 and VCAM-1 expression, HL60 adhesion to HUVEC monolayers, as well as the expression of CD11b/CD18 integrins on human leukocytes. Interestingly, although extracellular high D-glucose was not enough to induce the expression of endothelial CAMs, it significantly enhanced the induction of endothelial ICAM-1 and VCAM-1 elicited by IL-1β. Such a potentiating effect of high D-glucose was not restricted to IL-1β, as it was also observed for the pro-inflammatory cytokine TNF-α, whose levels are elevated in the circulation of diabetic patients and whose release to the circulation is promoted by hyperglycemia [4]. The *in vitro* adhesion of HL60 leukocytes to endothelial cells induced by IL-1β was also potentiated in a high D-glucose environment. It can therefore be concluded that high D-glucose on its own does not promote leukocyte-endothelial cell interactions, but rather plays a modulatory role in such events by enhancing an ongoing pro-inflammatory response on endothelial cells. In agreement with these observations, we have recently demonstrated that a pro-inflammatory preconditioning is required for high D-glucose to inflame human vascular smooth muscle [36]. Additionally, the fact that high D-glucose did not potentiate CD11b/CD18 integrins induction by IL-1β in human leukocytes suggests that the synergism between high D-glucose and pro-inflammatory cytokines may not affect every cell type involved in leukocyte recruitment to the vascular wall, but rather more specifically occurs in vascular cells.

We next aimed to gain insight into the cell signaling pathways mediating the synergistic action between D-glucose and IL-1β in human endothelial cells. Our data showed that just increasing the extracellular D-glucose concentration did not activate ERK 1/2 nor NF-κB in HUVEC. While this observation contrasts with previous reports showing ERK 1/2 and NF-κB activation in HUVEC by high D-glucose [14], [37], other studies have also failed to detect the activation of both molecules in endothelial cells exposed to elevated D-glucose levels [20], [38], [39]. We nevertheless observed that high D-glucose potentiated the activation of both ERK 1/2 and NF-κB elicited by IL-1β, indicating that the elevation of extracellular D-glucose results in an over-activation of endothelial signaling molecules triggered by a pro-inflammatory stimulus. In this context, we have recently shown that, in cultured human vascular smooth muscle cells, high D-glucose activates the ERK 1/2 - NF-κB - inducible nitric oxide synthase axis, but only when this signaling pathway is previously triggered by an exogenous inflammatory stimulus [36]. In agreement with others [40], [41], we have further found in the present work that ERK 1/2 and NF-κB activation was responsible for endothelial VCAM-1, but not ICAM-1, induction. Other signaling molecules, like poly(ADP-ribose) polymerase-1 (PARP-1) [42] and AP-1 [43] have been reported as promoters of ICAM-1 expression in different vascular cell types, and might therefore be involved in the induction of ICAM-1 by IL-1β observed herein and perhaps also over-activated by high D-glucose. Blocking VCAM-1 expression was not sufficient to totally prevent HL60 adhesion to endothelial cells, neither under low or high D-glucose conditions. Indeed, under NF-κB or ERK 1/2 blockade, the levels of ICAM-1 induced by IL-1β remained unchanged or even elevated, which may still facilitate HL60 adhesion. In this context, it is worth noting that the levels of lymphocyte function-associated antigen (LFA)-1 integrin (the counterpart for endothelial ICAM-1) in HL60 leukocytes are about 6-fold higher than those of very late antigen (VLA)-4 (the counterpart for endothelial VCAM-1) (unpublished observations).

The synergistic pro-inflammatory action between D-glucose and inflammatory stimuli may in fact be on the basis of the highly controversial reports existing on the ability of D-glucose *per se* to trigger endothelial cell activation *in vitro*. We propose that a pro-inflammatory response to high D-glucose can only occur when cultured cells are already in an inflammatory state. As cells in culture are in a rather artificial condition that facilitates inflammation, they are therefore easily primed to respond to abnormally high D-glucose. The degree of stress under which cultured cells are when used for experimentation is variable, which might explain the discrepancies encountered among the different studies.

The enhanced acute release of pro-inflammatory cytokines observed during postprandial hyperglycemia in diabetic patients [4] has been related with a higher risk of suffering cardiovascular events [4], [13]. Studies *in vitro* using human endothelial cells have also suggested that intermittent rather than constant high D-glucose levels can be more effective in inducing the expression of VCAM-1 and ICAM-1 [17]. By using intravital microscopy in the rat mesenteric microvasculature, Booth et al. [44] analyzed the effects of local acute intraperitoneal hyperglycemia on leukocyte-endothelial cell interactions *in vivo*. These authors reported increased leukocyte rolling and adherence after 12 h of intraperitoneal administration of moderate (20–25 mg/kg) and high (40–45 mg/kg) doses of D-glucose, while no effect was observed with the lowest dose (8–10 mg/kg) [44]. Herein, we have used a similar *in vivo* model of local hyperglycemia to analyze the influence of a high dose of D-glucose (40 mg/kg) on leukocyte trafficking after 18 h of its acute intraperitoneal injection. In accordance with our *in vitro* findings, IL-1β significantly increased leukocyte rolling flux, adhesion, and migration *in vivo*. More interestingly, D-glucose markedly enhanced all these parameters, therefore favoring vascular inflammation, but only when the cytokine was co-injected. In fact, and in contrast with the observations of Booth et al. [44], the mere administration of D-glucose in the absence of the cytokine did not alter leukocyte trafficking parameters. These functional data were accompanied by a parallel expression of both CAMs in the mesenteric microvasculature.

Taken together these results, we propose that acute hyperglycemia *per se* is not sufficient to promote vascular inflammation. More likely, the abnormally increased postprandial glucose concentrations observed in the metabolic syndrome and type 2 diabetes [45] can only exaggerate the effects of an ongoing inflammatory response. This may in fact constitute the main mechanism by which postprandial hyperglycemia promotes vascular inflammation and the development of diabetic vasculopathy [4], [13], [46]. Even more, such a synergistic effect between high D-glucose and inflammation could also explain why the expression of CAMs and other inflammation-related molecules in response to an infection is higher in hyperglycemic experimental models of diabetes [47].

The results obtained in the present study using from *in vitro* to *in vivo* approaches highlight that a pro-inflammatory pre-conditioning is necessary for extracellular high D-glucose to exert a deleterious effect in the vasculature. A recent study in human endothelial retinal cells suggests that cytokines, rather than high D-glucose, are responsible for diabetes-related retinal endothelial injury [38]. On the other hand, our data support the proposal that D-glucose by itself is likely not a major mechanistic factor in the development of diabetes-induced atherosclerosis [29] and they can provide a possible explanation for the failure of intensive blood-glucose control in preventing cardiovascular events associated to type 2 diabetes [26]–[28].

Our findings further underpin the relevance of the chronic low-grade pro-inflammatory environment observed in diabetes mellitus, as a pivotal factor conditioning the early pro-atherosclerotic actions of elevated extracellular D-glucose levels. In fact, they are in line with clinical observations in which a synergistic action of hyperglycemia and inflammation seems to be critical in clinical outcomes of acute coronary syndromes [48]. It is noteworthy that the inflammatory environment that occurs in type 2 diabetes is a rather complex phenomenon involving different cells and mechanisms and not strictly related to hyperglycemia [45]. Therefore, treating only hyperglycemia may be not sufficient to prevent and treat diabetic vasculopathy, and additional therapeutical goals, such as lowering chronic inflammation, should be carefully considered [49]. In this context, the subcutaneous administration of the recombinant human interleukin-1-receptor antagonist anakinra has recently been shown to reduce the markers of systemic inflammation in type 2 diabetic patients [45].

In conclusion, the present work provides an experimental basis to explain the failure of those therapeutic approaches directed only to reduce hyperglycemia in preventing diabetes-associated atherosclerosis and calls attention on the necessity to reduce systemic inflammation, either pharmacologically or through physical exercise [50], in order to increase the benefit in preventing cardiovascular events linked to type 2 diabetes or the metabolic syndrome.

## Supporting Information

Video S1 Confluent HUVEC monolayers were exposed to 5.5 mmol/L extracellular D-glucose for 18 h prior to leukocyte perfusion (1.5×106 cells/mL) drawn at 0.5 dynes/cm2 (x200).
(2.77 MB MOV)

Video S2 Confluent HUVEC monolayers were exposed to 22 mmol/L extracellular D-glucose for 18 h prior to leukocyte perfusion (1.5×106 cells/mL) drawn at 0.5 dynes/cm2 (x200).
(2.76 MB MOV)

Video S3 Confluent HUVEC monolayers were exposed to 5.5 mmol/L extracellular D-glucose in presence of IL-1β (5 ng/mL) for 18 h prior to leukocyte perfusion (1.5×106 cells/mL) drawn at 0.5 dynes/cm2 (x200).
(2.66 MB MOV)

Video S4 Confluent HUVEC monolayers were exposed to 22 mmol/L extracellular D-glucose in presence of IL-1β (5 ng/mL) for 18 h prior to leukocyte perfusion (1.5×106 cells/mL) drawn at 0.5 dynes/cm2 (x200).
(2.61 MB MOV)
